# Rapid Identification of Malaria Vaccine Candidates Based on α-Helical Coiled Coil Protein Motif

**DOI:** 10.1371/journal.pone.0000645

**Published:** 2007-07-25

**Authors:** Viviane Villard, George W. Agak, Géraldine Frank, Ali Jafarshad, Catherine Servis, Issa Nébié, Sodiomon B. Sirima, Ingrid Felger, Myriam Arevalo-Herrera, Socrates Herrera, Frederic Heitz, Volker Bäcker, Pierre Druilhe, Andrey V. Kajava, Giampietro Corradin

**Affiliations:** 1 Department of Biochemistry, University of Lausanne, Epalinges, Switzerland; 2 Pasteur Institute, Paris, France; 3 Centre National de Recherche et Formation sur le Paludisme, Ouagadougou, Burkina Faso; 4 Swiss Tropical Institute, Basel, Switzerland; 5 Institute of Immunology, University of Valle, Cali, Colombia; 6 CRBM, CNRS, University of Montpellier, Montpellier, France; National Institute of Allergy and Infectious Diseases, United States of America

## Abstract

To identify malaria antigens for vaccine development, we selected α-helical coiled coil domains of proteins predicted to be present in the parasite erythrocytic stage. The corresponding synthetic peptides are expected to mimic structurally “native” epitopes. Indeed the 95 chemically synthesized peptides were all specifically recognized by human immune sera, though at various prevalence. Peptide specific antibodies were obtained both by affinity-purification from malaria immune sera and by immunization of mice. These antibodies did not show significant cross reactions, i.e., they were specific for the original peptide, reacted with native parasite proteins in infected erythrocytes and several were active in inhibiting *in vitro* parasite growth. Circular dichroism studies indicated that the selected peptides assumed partial or high α-helical content. Thus, we demonstrate that the bioinformatics/chemical synthesis approach described here can lead to the rapid identification of molecules which target biologically active antibodies, thus identifying suitable vaccine candidates. This strategy can be, in principle, extended to vaccine discovery in a wide range of other pathogens.

## Introduction

Human *Plasmodium falciparum* (*Pf*) infection is a dramatic public health problem. Today approximately forty percent of the world's population is at risk of malaria. Malaria causes more than 300 million acute clinical cases, and at least one million deaths annually. Ninety percent of malaria deaths occur in sub-Saharan African countries mostly among young children and pregnant women (http://rbm.who.int). Thus, there is an urgent need of a malaria vaccine. However, vaccine discovery, in general and particularly in malaria, is still a very empirical process. In fact, protective antigens do not bear any structural, physico-chemical or sequence-related characteristics that would allow their identification. For protective humoral responses, the only recognized characteristics of antigens are their antigenicity/immunogenicity and accessibility. In addition, the lack of surrogate markers of protection renders the vaccine discovery process difficult and time consuming. Hence, in spite of a constant and impressive progress in molecular biology techniques and antigen identification and expression [1], [2], vaccine discovery is still labor-intensive, making the approach fastidious, costly and poorly adapted to high-throughput screening. Proper protein folding and solubility remain a limitation in numerous cases. Thus, overcoming the bottlenecks of manufacturing and identification of fragments/proteins, as possible targets of a protective immune response still constitute a scientific and technical challenge.

We addressed this challenge by combining bioinformatics, chemical peptide synthesis and functional protection assays. We focused on the search for α-helical coiled coil motifs that, in general, do not exhibit a folding problem, and are a target of effective antibodies for several current malaria vaccine candidates (eg. LSA-1 [3], LSA-3 [4], MSP-3 [5], and MSP-6 [6] and other pathogens [7]. MSP-1, another leading malaria vaccine [8], also contains predicted α-helical coiled coil regions (unpublished results). Our choice was based on the following considerations. First, the α-helical coiled coil motif bears a characteristic seven amino acid residue repeat (**abcdefg**)_n_ with hydrophobic residues located in **a** and **d** positions and hydrophilic residues generally elsewhere. This motif can be easily identified by bioinformatic analysis. Secondly, an important known characteristic of the α-helical coiled coil domains is that, taken separately from the whole protein, they frequently and readily fold into the same stable oligomeric structure [9]. Thirdly, for this reason, the α-helical coiled coil fragments are frequently recognized by conformational dependent antibodies, and can similarly elicit antibodies reactive with structurally “native” epitopes. In addition, these domains are short (about 40 residues) and can be rapidly produced by chemical synthesis. Furthermore, when the antibodies have an anti-parasite biological activity, this designates the corresponding proteins/fragments as potential, novel vaccine candidates to be further developed and assessed. Here, we focus on the *Pf* parasite erythrocytic stage, a target of protective antibodies and describe a straightforward, rapid procedure based on bioinformatic analysis of α-helical coiled-coil motifs and peptide synthesis.

## Results and Discussion

The screening of the *Pf* genome [10] using generalized sequence profiles [11] identified several hundred proteins containing putative α-helical coiled coil motifs. Through proteome and transcriptome data [12]–[14] we assessed which of these molecules are expressed in the *Pf* parasite erythrocytic stage. The combined analysis/assessment identified over 100 segments associated with this stage and displaying the putative α-helical coiled coil motifs with high probability score (Table S1). Out of these α-helical coiled coil fragments, in general 30–40 amino acids long, present either in the same protein or in different ones, 95 were chemically synthesized and HPLC purified. Among them, longer peptides (up to 70 amino acids), which contained one or more α-helical coiled coil domains, were also synthesized (antigenS 1, 12 and 83; Table S1). The selected antigens were then tested in ELISA assays for reactivity with three panels of sera obtained from adult donors from Burkina Faso, Tanzania and Colombia, respectively. To our surprise, all of the α-helical coiled coil fragments were antigenic, though the prevalence of responders varied greatly (Tables 1 and S1). In this manner, 71 proteins were identified whose lengths varied from 200 to 10,000 amino acids. Twenty-one peptides with the highest prevalence of responders and ELISA mean OD value were selected for further studies. Variation in recognition among the three panels of sera may be due to differences in the genetic background of the hosts, of the parasites and, most likely, to distinct malaria transmission conditions in the three regions. The high level of recognition of the α-helical coiled coil motifs may be explained by the fact that taken separately from the whole protein these fragments readily fold into the same stable structure in aqueous solution.

**Table 1:** Antibody response and ADCI activity.

Peptides	Proteins	Sequences	%^1^>control+3 SD	Ratio^2^ (%)	Mean OD	%^1^ >control+3 SD	Ratio^2^ (%)	Mean OD	%^1^ >control+3 SD	Ratio^2^ (%)	Mean OD	SGI^3^ (%)
			Burkina Faso sera			Tanzanian sera			Colombian sera			
**8**	PFB0145c	IKTMNTQISTLKNDVHLLNEQDKLNNEKGTLNSKISELNVQIMDL^5^	70	43	0.294	36	26	0.274	15	5	0.101	0
**9**		LLSKDKEIEEKNKKIKELNNDIKKL	32	8	0.194	56	40	0.258	26	10	0.132	48
**11**		ICSLTTEVMELNNKKNELIEENNKLNLVDQGKKKLKKDVEKQKKEIEKL	54	32	0.285	40	12	0.456	28	28	0.156	N D^4^
**12**		VDKIEEHILDYDEEINKSRSNLFQLKNEICSLTTEVMELNNKKNELIEENNKLNLVDQGKKKLKKDVEKQKKEIEKL	65	41	0.242	74	45	0.307	26	23	0.174	50
**13**		LDENEDNIKKMKSKIDDMEKEIKYR	27	27	0.214	38	24	0.149	64	26	0.170	37
**14**	PFC0245c	GMNNMNGDINNIN(GDINNMN)4	41	30	0.266	40	21	0.185	44	31	0.215	71
**27**	MAL6P1.37	KKRNVEEELHSLRKNYNIINEEIEEIT	54	30	0.265	69	33	0.237	18	8	0.124	106
**45**	PF11_0207	EEIKEEIKEVKEEIKEVKEEIKEVKEEIKEVKEEIKE	70	57	0.554	62	48	0.263	59	36	0.295	36
**50**	PFL1605w	KNDINVQLDDINVQLDDINVQLDDINIQLDEINLN	43	32	0.352	75	43	0.220	21	8	0.111	N D
**52**	PFL0770w	KIQIEEIKKETNQINKDIDHIEMNIINLKKKIEF	51	24	0.180	36	24	0.187	26	13	0.108	19
**54**	MAL6P1.147	DSMNNHKDDMNNYNDNINNYVESMNNYDDIMNK	59	43	0.288	76	36	0.260	31	18	0.199	0
**66**	PFL0250w	MCELNVMENNMNNIHSNNNNISTHMDDVIE	51	32	0.280	74	31	0.281	56	28	0.164	56
**72**	PFC0760c	KEIQMLKNQILSLEESIKSLNEFINNLKN	30	11	0.127	67	45	0.201	26	3	0.103	N D
**76**	MAL13P1.304	GGLKNSNHNLNNIEMKYNTLNNNMNSINK	57	27	0.353	50	36	0.259	51	10	0.136	35
**77**	PF08_0048	EKLKKYNNEISSLKKELDILNEKMGKCT	54	43	0.309	79	4	0.359	51	36	0.192	36
**79**	PFB0315w	EKMNMKMEQMDMKMEKIDVNMDQMDVKMEQMDVKMEQMDVKMKRMNK	76	51	0.176	93	65	0.406	54	13	0.109	16
**80**	MAL8P1.12	KNKLNKKWEQINDHINNLETNINDYNKKIKEGDSQLNNIQLQCENIEQKINKIKE	89	57	0.297	80	48	0.246	33	5	0.101	45
**81**	PF07_0086	NEMNKEVNKMNEEVNKMNEEVNKMNEEVNKMNKEVNKMDEEVNKMNKEVNKMNK	89	51	0.352	68	55	0.378	15	21	0.100	0
**83**	PFC0345w	QNKMENDMNIIKNDMNIMENDMNIMENDMNIIKNDMNIMEKDMNIIKNDMNIIKNNMNIIKNEMNIIKNV	51	46	0.302	75	73	0.505	15	5	0.107	0
**90**	PFD0520c	TKKLNKELSEGNKELEKLEKNIKELEETNNTLENDIKV	59	41	0.401	88	53	0.406	28	10	0.128	43
**94**	PFD0970c	ENINNMDEKINNVDEQNNNMDEKINNVDEKK	43	43	0.200	78	50	0.319	10	3	0.107	0

1 % of positive responses evaluated as OD values higher than the mean negative control+3SD.

2 OD ratio higher than 2 between the mean duplicate experimental and mean negative control OD.

3 Specific Growth Inhibitory Index

4 Not Done

5 A, alanine; C, cysteine; D, aspartic acid; E, glutamic acid; F, phenylalanine; G, glycine; H, histidine; I, isoleucine; K, lysine; L, leucine; M, methionine; N, asparagine; P, proline; Q, glutamine; R, arginine; S, serine; T, threonine; V, valine; W, tryptophan; Y, tyrosine

Indeed, circular dichroism (CD) studies of selected peptides associated with biological activities (Tables 1 and 2) indicate that they predominantly assume an α-helical conformation in water. Peptides 14, 27 and 45 (Figure S1A) exhibit a CD pattern characteristic of a high α-helical content, whereas the remaining peptides show CD profiles similar to that shown for peptide 12 (Figure S1B) or intermediate between those shown in Figures S1A and S1B characteristic of a partial α-helical organization. When analyzed by size exclusion chromatography on FPLC columns, peptides presented elution profiles between those exhibited by chymotrypsin and ribonuclease (MW 24 and 13kDa, respectively). The CD and size exclusion chromatography results suggest that peptides adopt an α-helical coiled-coil structure, which need to be unambiguously ascertained by NMR and ultra-centrifugation studies.

**Figure 1:**
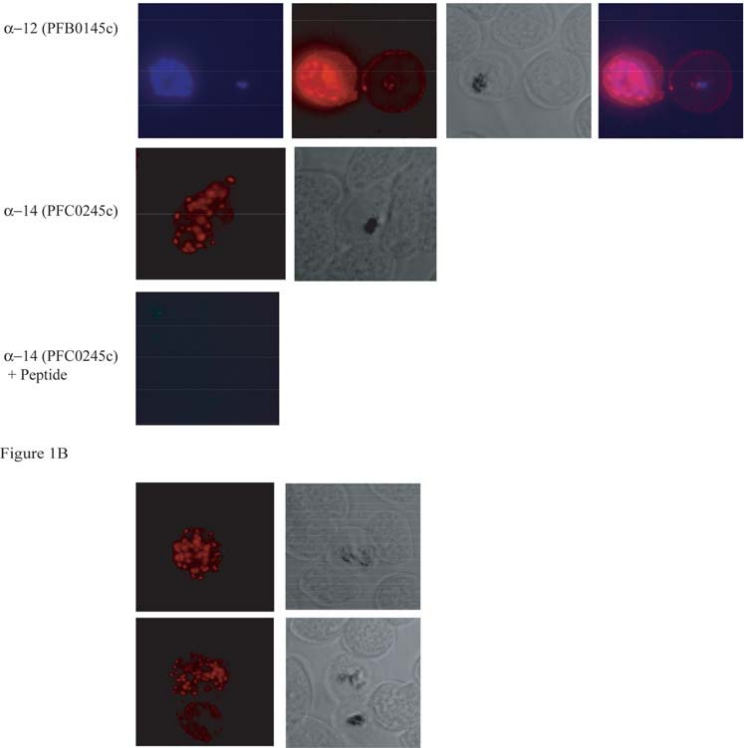
Immunofluorescence microscopy analysis of *Pf* 3D7 parasites with peptide specific antibodies. Acetone/methanol-fixed schizonts and merozoites were reacted with A: human peptide specific, affinity purified antibodies obtained with peptides 12 and 14 (Table 1) and B: sera from mice immunized with peptide 27 (Table 1). Grey: bright field images; blue staining: indicates DAPI nuclear staining of schizont stage parasites; red staining shows labeling of peptide specific antibodies by Cy3-conjugated anti-human or anti-mouse IgG specific antibody. Merge picture is an overlay of the blue and red fluorescence channel.

To test the biological activity of peptide-specific antibodies, the latter were purified by affinity chromatography using three serum pools obtained from Papua New Guinean adults. The 3 serum pools were first tested in ELISA assays against 21 peptides that were the most antigenic (Table 1); from these, 18 peptide-specific antibodies were purified from the most positive serum pool and tested again in ELISA. These 18 antibodies all reacted with parasite native proteins in infected red blood cells as shown by IFAT (Figure 1A; Table 2). Reactivity was restricted to blood stages, since the antibodies did not react with sporozoites stages (data not shown), and this reactivity was also peptide-specific as shown by IFAT competition assays with the corresponding peptide (Figure 1A).

**Table 2:** Summary data of human and mouse antibodies.

Peptides	Proteins	Ab titer	IFAT (mouse Ab)^1^		Prevalence^2^		IFAT (Human Ab)^3^		SGI^4^ (%)
			Rings	Schizonts	BF (%)	Tz (%)	Trophozoites	Schizonts	
**8**	PFB0145c	8100–72900	-	-	70	36	+	+	0
**9**		100-100	-	-	32	55	+	+	48
**12**		218700-218700	-/+	+	65	74	+	+	50
**13**		100-100	-	-	27	38	+	+	37
**14**	PFC0245c	2700-24300	-	-/+	41	40	+	+	71
**27**	MAL6P1.37	8100-24300	+	+	54	69	+	+	106
**45**	PF11_0207	100-100	-	-/+	70	62	+	+	36
**52**	PFL0770w	2700-8100	-	-	51	36	+	+	19
**54**	MAL6P1.147	100-300	N D 5	N D	59	76	+	+	0
**66**	PFL0250w	900-8100	-	-	51	74	+	+	56
**76**	MAL13P1.304	100-100	-	-	57	50	+	+	35
**77**	PF08_0048	8100-218700	-	-	54	79	+	+	36
**79**	PFB0315w	8100-218700	-/+	-/+	76	93	+	+	16
**80**	MAL8P1.12	72900-218700	+	+	89	80	+	+	45
**83**	PFC0345w	72900-218700	+	+	51	75	+	+	0
**90**	PFD0520c	100-2700	-	-	59	88	+	+	43

1 Mouse antibodies were used at a dilution of 1:100

2,4 For easier comparison, data are duplicated from Table 1. BF, Burkina Faso; Tz, Tanzania.

3 Human purified antibodies were used at 5 µg/mL; IFAT was not performed on ring stages.

5 Not Done

**Figure 2:**
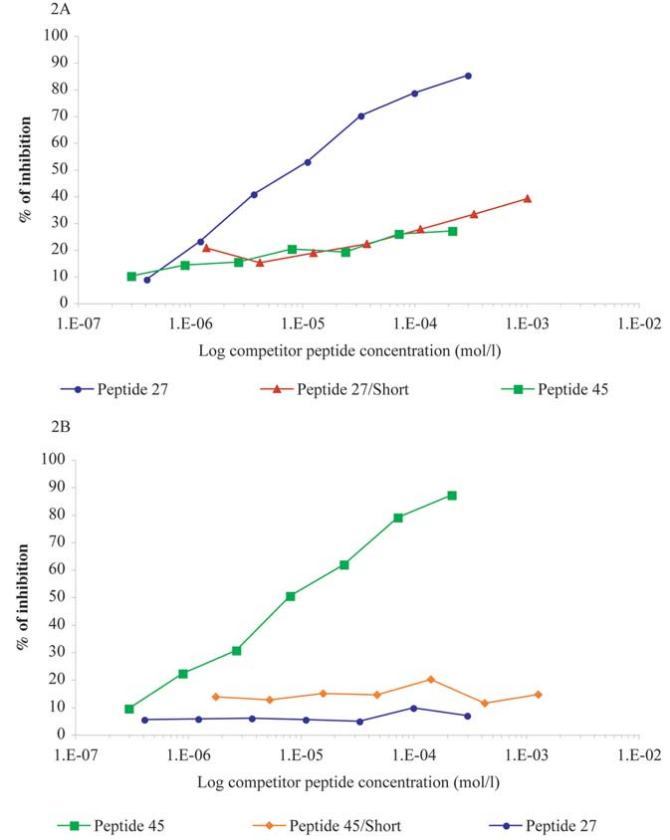
ELISA inhibition assay using anti-human peptide specific antibodies. Binding of peptide specific antibodies to peptides 27 (A) and 45 (B) absorbed on ELISA plates was inhibited by incubating specific antibodies (1–2 µg/ml) with peptides 27 or 45 or shorter fragments of the two, designated as 27/short (NEEIEEIT) or 45/7 short (KEEIKE) (see Material and Methods).

The specificity of the antibodies obtained was investigated in detail, particularly since several peptides contain glutamic acid (Glu)-rich sequences which are known to generate cross reactivity among several malarial Glu-rich proteins [15]. Cross-reactions were systematically investigated using each of the 18 affinity-purified antibodies on each of the 18 peptides. Results show that -with few exceptions- each antibody preferentially recognizes the peptide against which antibodies were affinity-purified, i.e. they are specific for the corresponding peptide (Table S2). To determine if non-specific antibody binding to solid phase-adsorbed antigens could be responsible for the rare cross-reactivities detected, ELISA competition assays were performed. To this end, binding of antibodies to the solid phase-adsorbed antigen was competed against increasing concentrations of the homologous or cross-reacting peptides. Only homologous peptides competed best whereas peptides having sequence similarity did not (Figures 2A, 2B, and S2B), or at a much higher concentration (Figure S2A) including the shorter Glu-rich peptides derived from the C-terminus of peptide 27 and 45 (Figures 2A and 2B). Finally, the pattern of recognition of peptides by the various sera tested, which differ markedly from one to the other (data not shown), confirms the above results i.e., specificity of antibodies to the corresponding peptide.

Antibodies corresponding to the 18 selected peptides were tested for direct and cell-mediated anti-parasite activity. Clinical experiments have shown that the Antibody-Dependent Cell-mediated Inhibition (ADCI) of *P. falciparum* malaria represents one of the mechanisms controlling parasitemia and thereby clinical manifestations in humans [16]. Twelve peptide-specific antibodies proved able to induce a strong (more than 40%) and intermediate (lower than 40%) monocyte-dependent parasite killing (Table 1), whereas, in the absence of monocytes, no direct effect of antibodies on parasite growth was observed. The effects were in the range observed with antibodies from African adults who have the highest natural protection known against malaria. Therefore peptide-specific, human affinity-purified antibodies were functionally effective as shown by their ability to react with parasite proteins and to inhibit parasite growth. Thus, *in vitro* functional assays show that peptide-specific antibodies elicited by natural exposure to the parasite can induce protective mechanisms effective against malaria.

Sixteen peptides -twelve targeted by ADCI positive antibodies and four controls-were used to immunize CB6F1 mice (Table 2). Eleven of them elicited an intermediate or high antibody response, four of which also recognized the parasite protein in infected erythrocytes as determined by IFAT (Figure 1B; Table 2). As seen before for human antibodies, recognition was restricted to blood stages since sporozoites were negative in IFAT assays (data not shown) and by IFAT competition assays with the corresponding peptide (Figure 1B). Anti-peptide 27 mouse antibodies, which are positive in IFAT, are also specific for the homologous peptide 27 but not for the sequence related peptides 9, 12 and 45 (Table S2). Thus, peptides, which were chosen for their propensity to form α-helical coiled coil, can induce the production of antibodies that recognize epitopes present in the native protein. Improvement of the immunogenicity and structural specificity of the remaining peptides might be achieved in the future by a) a short elongation at the N- and C- terminal ends, b) stabilizing the α-helix as suggested by Cooper et al. and Lu and Hodges [17], [18] and/or c) use of other adjuvants.

Genetic polymorphism in current vaccine candidates is a major limitation to vaccine development. However available genotyping studies of our peptide sequences in parasite isolates of worldwide origin indicate very limited polymorphism (PlasmoDB 5.2, and unpublished sequencing data). A few peptide DNA sequences show deletion of one entire heptad repeat so that the shorter region still preserves its potential for the α-helical coiled coil formation.

With regard to the structural features and cellular location prediction of the proteins corresponding to the peptides selected for ADCI assays (Table 1), 15 of the proteins contain a pentapeptide conforming to the PEXEL consensus [19, 20; 21, 22], but that none of these have a position within the amino acid sequence that conforms to the location of known active PEXEL motifs (see Materials and Methods and Table S3); 12 have trans-membrane segments, and none of them has a GPI anchor. Only one protein contains a signal sequence. Fourteen proteins are predicted to be in the cytoplasm, one in the nucleus, one in the mitochondria, and one in the peroxysomes (Table S3). The prediction of the sub-cellular localization of these proteins should be taken with caution because gene annotation is being constantly updated and/or protein trafficking of the parasite is complex and not fully elucidated [23].

Further investigations will be required to determine the actual localization of the corresponding antigens. The predicted localization is a priori surprising for molecules able to trigger an ADCI activity. However, recent studies have shown that in addition to merozoite surface proteins, soluble proteins released at the time of schizont rupture were equally effective at triggering ADCI provided they defined at least two epitopes [24], which is the case for α-helical coiled coil heptad repeats. Therefore, molecules expressing a trans-membrane domain that can be exported to the parasite or host cell membrane, as well as molecules present in the cytoplasm of maturing schizonts and released by bursting schizonts can trigger antibodies to cross-link Fc-γ receptors on monocytes to achieve *Pf* parasite killing.

In conclusion, an approach combining a genome-wide search by bioinformatics of α-helical coiled coil protein motifs and chemical synthesis can lead to the rapid identification and development of new malaria vaccine candidates. In fact, this approach is straightforward and easy to scale up; vaccine formulations may comprise mixtures of peptides or single constructs made up of several epitopes. In principle, this strategy can be extended to the discovery of proteins and vaccine candidates in other complex pathogens.

## Materials and Methods

### Bioinformatics screening

The *Pf* 3D7 genome [10] was used for the bioinformatics analysis. The generalized sequence profile method and the pftools package [11] were used to search for the short α-helical coiled coil domains. The coiled coil profiles were constructed using an alignment of several amino acid sequences corresponding to the known coiled coil domain. Two profiles containing four and five heptad repeats were used for the analysis. The cut-off levels of the profiles were chosen by tests performed against sequence database of proteins with the known 3D structures. Subsequently, the coiled coil regions selected by this approach were tested manually for the presence of the characteristic heptad repeats. These proteins were also analyzed by the COILS program [25].

The selected α-helical coiled coil containing proteins were further tested on their possible surface location and GPI anchoring by using the following programs: identification of potential signal peptides, SecretomeP and SignalP (http://www.cbs.dtu.dk/services/) [26]; transmembrane spanning regions (TMPRED http://www.ch.embnet.org/software/TMPRED_form.html and TMHMM http://www.cbs.dtu.dk/services/TMHMM; [27], [28]), and GPI-anchored proteins (http://mendel.imp.univie.ac.at/sat/gpi/gpi_server.html; [29]) and prediction of sub-cellular localization (pTARGET http://bioinformatics.albany.edu/∼ptarget; [30]). To identify the PEXEL-like motifs in sequences of the selected proteins we used the following pattern [KR][GAVLIMFWPSTCYNQ][LIA][GAVLIMFWPSTCYNQ] [DEQ] that represents a combination of the PEXEL patterns indicated in recent papers [21], [22]. The presence of the identified proteins in the asexual erythrocytic stages was also checked using the published data on the transcriptome and proteome of this stage of development of *P. falciparum* (www.PlasmoDB.org; [31]).

### Peptide synthesis

Peptides were synthesized on the Advanced ChemTech (Hatley St George, UK) AC T348 Omega multi channel synthesizer and the Applied Biosystem synthesizer 431A and 433A (Foster City, CA) using solid-phase Fmoc chemistry. Crude peptides were purified by RP-HPLC (C18 preparative column) and analyzed by mass spectrometry (MALDI-TOF; Applied Biosystem, Foster City, CA). Chemicals and solvents used for peptide synthesis were purchased from Fluka (Buchs, Switzerland) and Novabiochem (Laufelfinger, Switzerland).

### Circular Dichroism Studies

Circular dichroism (CD) spectra of peptides were recorded on a JASCO J-810 spectrometer (JASCO corporation, Tokyo, Japan) equipped with a temperature controller and a 0.1 cm path length cuvette. The measurements were made in water at pH 7.3 and 22°C and at a peptide concentration of 0.2 mg/ml.

### Human Sera

The sera from Burkina Faso were collected in the village of Goundry located in the central Mossi Plateau, between 15 and 50 km north of the capital Ouagadougou, in the province of Oubritenga. The climate is characteristic of areas of Sudanese savannah, with a dry season from November to May and a rainy season from June to October. Malaria transmission is very high during the rainy season and markedly seasonal. Ethical clearance was obtained from the Ministry of Health, Burkina Faso. After obtaining informed consent from parents and caretakers, heparinized venous blood samples were collected during a cross-sectional survey during the malaria low transmission season 1998.

The Tanzanian sera came from a large-scale community based study undertaken in Kikwalila village, Kilombero District, Morogoro Region from 1982 to 1984. Blood samples from adults (>15 years) were taken by finger prick and the serum was kept at –70°C until use. Research and ethical clearance for the study was obtained by the Tanzanian Commission for Science & Technology.

The Colombian sera were collected in Buenaventura the main port on the Colombian Pacific Coast after human informed consent, during a cross sectional survey carried out from February to May 2002 within the framework of a project supported by the Colombian Research Council, COLCIENCIAS. The area has unstable transmission of both *P. falciparum* and *P. vivax* malaria. Ethical clearance to draw blood from human volunteers was obtained from the Institutional Review Board of Universidad del Valle. Blood was taken by venipuncture into tubes containing EDTA and sera fractionated and stored frozen until use.

The sera from adults from Papua New Guinea (PNG) pooled for affinity purification were collected in the Maprik district of the East Sepik Province, during a cross sectional survey in July 1992 within the framework of the Malaria vaccine Epidemiology and Evaluation Project (MVEEP) supported by the United States Agency for International Development [32]. The area is highly endemic for malaria. Ethical clearance for MVEEP was obtained from the PNG Medical Research Advisory Committee. Blood was taken by venipuncture into tubes containing EDTA.

The pool of immune African globulins (PIAG) used for ADCI was prepared from immune individuals living in endemic areas and negative control IgG (N-IgG) was obtained from a pool of more than 1000 French adult donors with no history of malaria. Briefly, the IgG fractions from both positive and negative controls were purified using a size exclusion Trisacryl® GF05M (Pall BioSepra®; Pall life Sciences, NY) column followed by an ionic exchange DEAE Ceramic HyperD® F column (Pall BioSepra®). Purified IgG were then extensively dialyzed against RPMI and kept at 4°C until use.

### Mouse immunization

CB6F1 mice were injected 3 times with 20 µg of the indicated peptide in Montanide ISA 720 at the base of the tail on day 1, 22 and 78. Bleeding was performed 10 days after the second and third immunization.

### ELISA

ELISA was performed according to Lopez et al. [33] and anti human IgG-or anti mouse IgG conjugated to alkaline phosphatase was used (Sigma, St Louis, MO) as second antibody. Individual human sera from 37, 42 and 39 adults donors from Burkina Faso, Tanzania and Colombia respectively were used at 1:200 dilution. Serum was considered positive if the optical density (OD) reading was higher than the mean OD value+3 standard deviation (SD) of the negative controls (individual serum samples from 8 to 11 naïve Swiss donors) or if the OD ratio of the mean of duplicate experimental values to the mean OD of the negative control was higher than 2. For mouse sera, the end point value was determined as the last dilution of the mean OD value+3 standard deviation (SD) of the negative control (non immune sera).

### ELISA Competition Assays

ELISA competition assays were performed by incubating either each of the 18 selected human affinity-purified antibodies, or antibodies elicited in mice, together with each of the 18 antigens over the indicated range of concentrations for 30 minutes at room temperature prior to addition to the ELISA peptide-coated plate wells.

### Antibody Purification by Affinity Chromatography

Antigen-Sepharose conjugate preparation: 5 mg of antigen was dissolved in 1 mL of coupling buffer (0.1 M NaHCO_3_ containing 0.5 M NaCl, pH 8.0). The CNBr-sepharose 4B (Amersham Bioscience AB, Uppsala, Sweden) was activated by swelling in 1 mM HCl and then washed with coupling buffer. The antigen solution was added to the gel and the mixture was stirred for 1h at RT. After the coupling reaction, excess antigen was washed away with coupling buffer. The unreacted activated groups were blocked by treatment with ethanolamine (0.25 M; pH 8.0) for 30 min at RT. The gel was then washed with sodium acetate buffer (0.1 M; pH 4.0), followed by coupling buffer. The antigen-sepharose beads were either used or stored at 4°C in PBS (1x) containing 1 mM azide.

Isolation of specific antibody: Pooled human serum was diluted five times with PBS (1x) containing 0.5 M sodium chloride and mixed with antigen-sepharose conjugate. This mixture was then stirred gently on a wheel O/N at 4°C. After centrifugation the supernatant was collected and stored at −20°C for further use. The antigen-sepharose beads were then washed with 5 mL of trizma base TRIS (20 mM containing 0.5 M NaCl, pH 8.0) then with 5 mL of TRIS (20 mM, pH 8.0). The elution of bound antibody was achieved with glycine (0.1 M, pH 2.5). The fractions obtained were instantly neutralized with TRIS (1 M, pH 8.0), dialyzed against phosphate buffer (0.1M, pH 7.0) and the antibody concentration was determined by the absorbance of the solution at 280 nm.

### Indirect Fluorescence Antibody Test (IFAT)

Slides coated with *Pf* sporozoites were dried at RT for 30 minutes, fixed with 100% acetone at 4°C for 10 minutes, washed 2 times in PBS-0.05% Tween 20, dried carefully and blocked with 20 µL/well of PBS-3% bovine serum albumin (BSA) for 30 minutes at RT. Slides coated with *Pf* merozoites were fixed with 100% acetone at −20°C for 15 minutes and dried O/N at RT. The appropriate antibody or serum dilutions prepared in PBS-3% BSA were distributed (10 µL/well) and incubated for 1h at RT in a humid chamber. After washing with PBS-0.05% Tween-20, goat anti-human or goat anti-mouse polyvalent immunoglobulins conjugated to Cy3 (Molecular Probes) diluted 1/500 in PBS-3% BSA or anti-human IgG (Fc specific) FITC conjugate (Sigma) diluted 1/50 in Evans blue solution (1/50000) was added (400 µL/slide) and incubated for 1h at RT in a humid chamber in the dark. Slides were washed as above, covered with 50 % glycerol, sealed and read using a fluorescence microscope (Leica DMIRB DC200).

### Parasites

The Uganda Palo Alto strain (FUP/C) was cultured in RPMI-1640 supplemented with 0.5% albumax I (GibcoBRL-Invitrogen, San Diego, CA). For ADCI assays, blood stage parasite cultures were synchronized by at least two successive sorbitol treatments followed, after maturation over 24 h, by floatation on 1% porcine skin gelatin type A (Sigma).

### Preparation of human blood monocytes

Blood monocytes (MN) were prepared from cytapheresis samples obtained from healthy blood donors with no previous history of malaria (Lecourbe Blood Bank, Paris, France). Peripheral blood mononuclear cells (PBMC) were separated on Ficoll density gradients J PREP (TechGen, Les Ulis, France) and washed in Ca^2+^ and Mg^2+^ free HBSS buffered with 10 mM HEPES (both from GibcoBRL-Invitrogen). Cells were then distributed on polystyrene 96-well flat-bottomed culture plates (TPP, Trasadingen, Switzerland) and adherent MN were selected by incubation for 2 h at 37°C, in a humidified 5% CO_2_ atmosphere. More than 90% of the adherent cells obtained in this manner were MN as estimated by the non-specific esterase test (α-naphtyl acetate esterase; Sigma). MN from each donor were tested prior to ADCI assays and only those without direct inhibitory effect were used in assays.

### ADCI *in vitro* assay

To wells containing 2×10^5^ MN purified as described above, 50 µl of an asynchronous parasite culture at 0.5 % parasitemia and 4 % hematocrit were added. Wells were then supplemented with test or control antibodies (Ab) and the total volume adjusted to 100 µl with culture medium. After 48 h and 72 h, 50 µl of culture medium were added to each well and after 96 h the ADCI assay was stopped and the final parasitemia was determined by light microscopy on Giemsa-stained smears by counting ≥50,000 red blood cells. For each Ab tested, duplicate wells included the following controls 1) non-specific monocytic inhibition, both MN+parasite, and MN+N-IgG+parasites and 2) direct inhibition by control or test IgG, both N-IgG+parasites, and test Abs+parasites. PIAG and N-IgG were used at a final concentration of 1 mg/ml as positive and negative controls respectively. Immunopurified tests Abs were used at 15 µg/ml. The Specific Growth inhibitory Index (SGI) which considers the parasite growth inhibition due to the effect of test Abs cooperating with MN was calculated as follows: SGI = 100×[1−(% parasitemia with MN and test Abs/% parasitemia test Abs)/(% parasitemia with MN and N-IgG/% parasitemia N-IgG)].

## Supporting Information

Figure S1 CD spectra of the peptides 45 (S1A) and 12 (S1B)
(12.32 MB TIF)

Figure S2 ELISA inhibition assay using anti-human peptide specific antibodies. Binding of peptide specific antibodies to peptides 76 (S2A) and 9 (S2B) absorbed on ELISA plates was inhibited by incubating specific antibodies (1-2 µg/ml) with peptides 14, 76 and 81 (S2A) and peptides 8 and 9 (S2B), respectively (see Material and Methods). Peptides 14, 76 and 79 share NNM or MNN as sequence similarity while peptides 8 and 9 do not exhibit any apparent sequence similarity.
(24.76 MB TIF)

Table S1 Antibody response.
(0.16 MB DOC)

Table S2 Cross-reactivity determination in ELISA among peptides tested in ADCI. The number in boxes represent the ratio between the experimental and control ELISA OD.
(0.36 MB DOC)

Table S3 Structural feature and cellular location prediction of the proteins containing the peptides whose specific antibodies were tested in ADCI (Table 1).
(0.05 MB DOC)
