# Supplementary figures and images for: Rapid Identification of Malaria Vaccine Candidates Based on α-Helical Coiled Coil Protein Motif
Source: PLoS One. 2007 Jul 25;2(7):e645. doi: 10.1371/journal.pone.0000645 (PMC1920550; doi:10.1371/journal.pone.0000645)

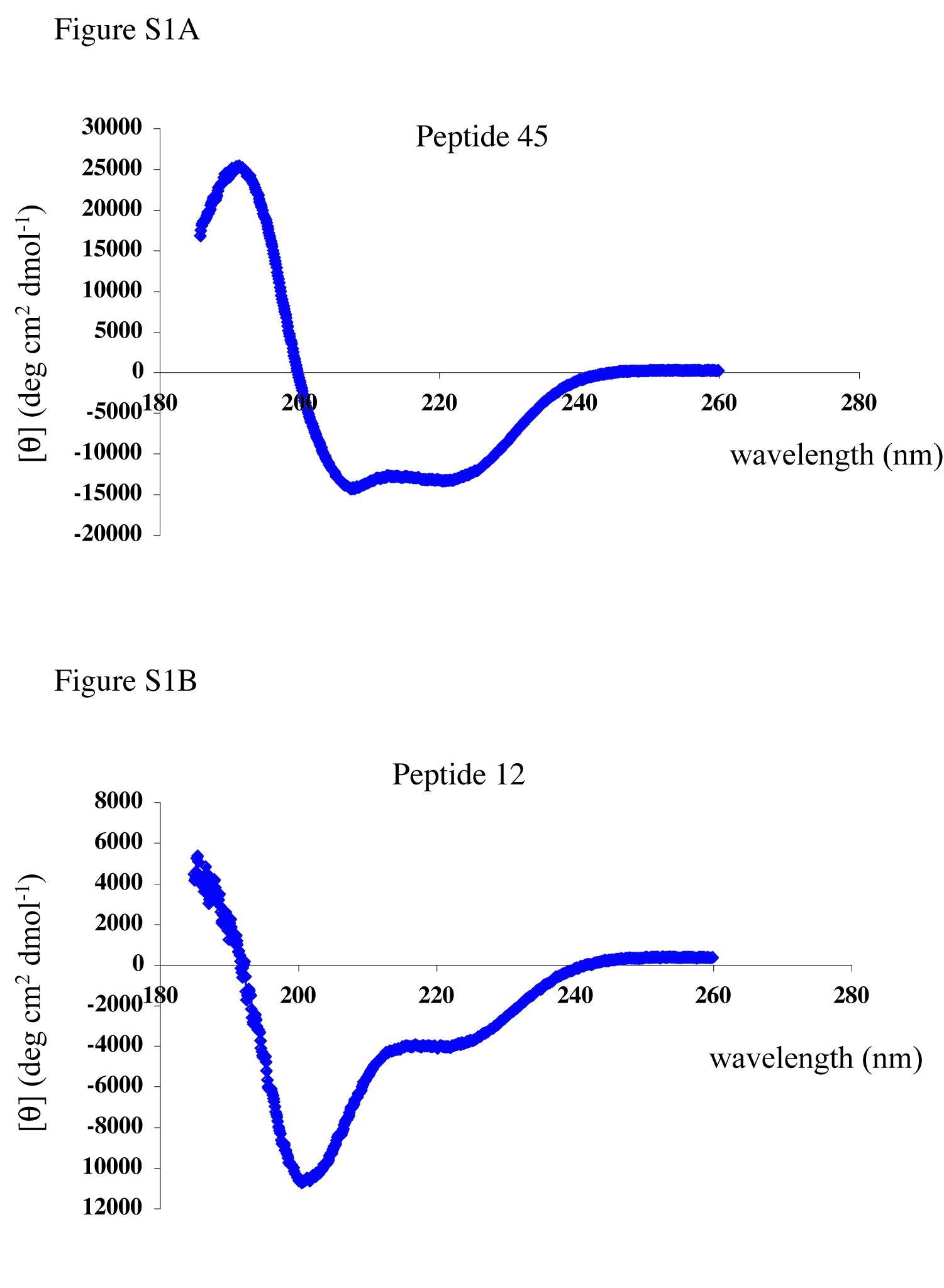

Supplement: Figure S1 — CD spectra of the peptides 45 (S1A) and 12 (S1B) (12.32 MB TIF) [file pone.0000645.s001.tif]

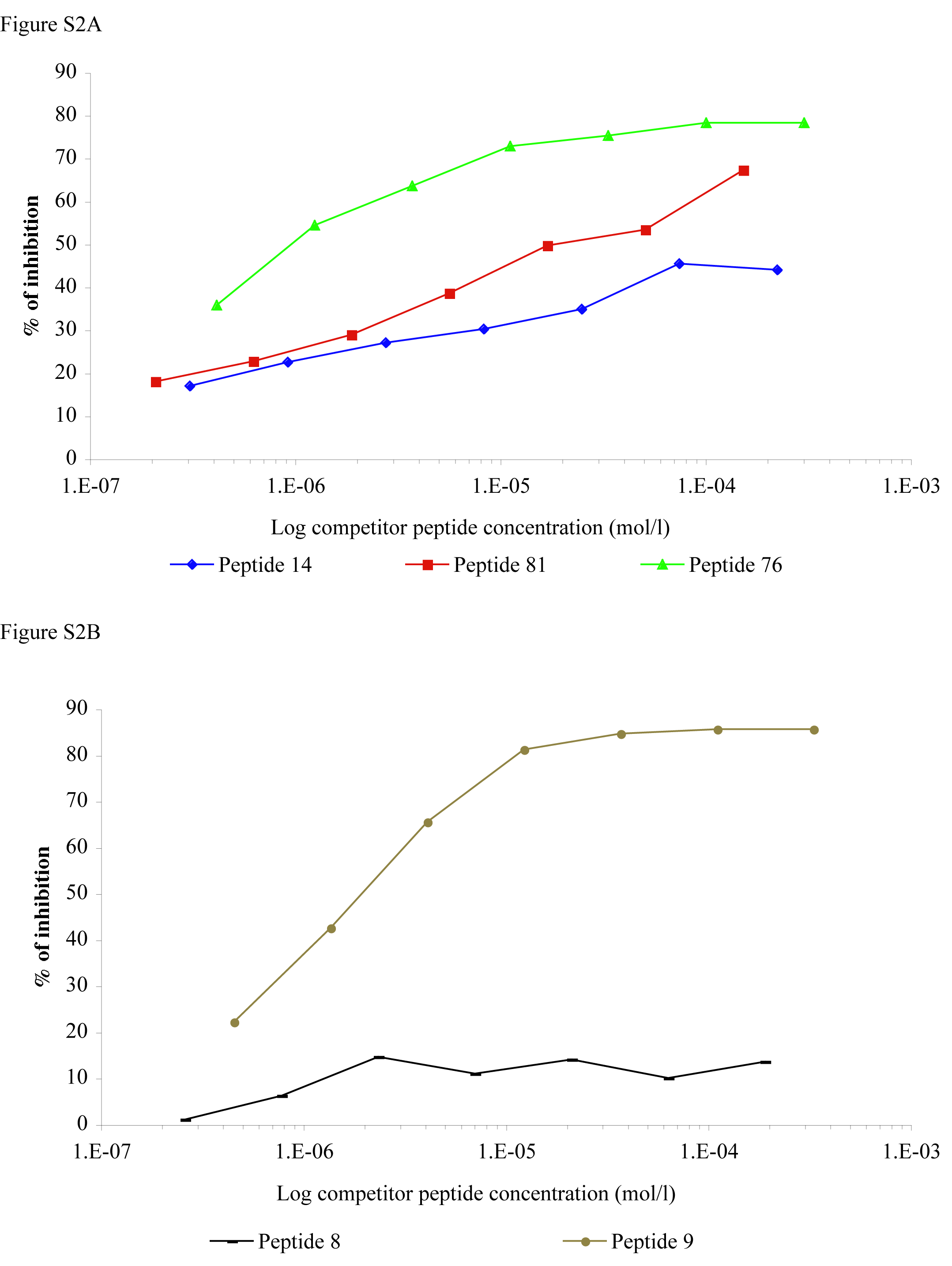

Supplement: Figure S2 — ELISA inhibition assay using anti-human peptide specific antibodies. Binding of peptide specific antibodies to peptides 76 (S2A) and 9 (S2B) absorbed on ELISA plates was inhibited by incubating specific antibodies (1-2 µg/ml) with peptides 14, 76 and 81 (S2A) and peptides 8 and 9 (S2B), respectively (see Material and Methods). Peptides 14, 76 and 79 share NNM or MNN as sequence similarity while peptides 8 and 9 do not exhibit any apparent sequence similarity. (24.76 MB TIF) [file pone.0000645.s002.tif]
