# Supplementary material for: Rapid Identification of Malaria Vaccine Candidates Based on α-Helical Coiled Coil Protein Motif
Source: PLoS One. 2007 Jul 25;2(7):e645. doi: 10.1371/journal.pone.0000645 (PMC1920550; doi:10.1371/journal.pone.0000645)
Supplement: Table S1 — Antibody response. (0.16 MB DOC) [file pone.0000645.s003.doc]

| Peptides | **Proteins** | Sequences | **%^1^** | **Ratio^2^**  **(%)** | **Mean OD** | **%^1^** | **Ratio^2^**  **(%)** | **Mean OD** | **%^1^** | **Ratio^2^**  **(%)** | **Mean OD** |
| --- | --- | --- | --- | --- | --- | --- | --- | --- | --- | --- | --- |
|  |  |  | **Burkina Faso sera** | | | **Tanzanian sera** | | | **Colombian sera** | | |
| **1** | PFA0170c | (VNNLDSTVNYMNSTGNNINNI)3^4^ | 14 | 3 | 0.168 | 38 | 10 | 0.154 | 18 | 8 | 0.151 |
| **2** | PFB0145c | TISSLSNKIVNYESKIEELEKELKEVK | 27 | 5 | 0.135 | 100 | 55 | 0.252 | 28 | 5 | 0.118 |
| **3** |  | NIKTMNTQISTLKNDVHLLNEQIDKLN | 27 | 5 | 0.146 | 48 | 7 | 0.109 |  |  |  |
| **4** |  | EIEKLNKQLTKCNKQIDELNEEVEKLN | 16 | 16 | 0.254 | 40 | 12 | 0.114 | 21 | 5 | 0.118 |
| **5** |  | IIDIKKHLEKLKIEIKEKKEDLENL | 41 | 32 | 0.236 | 43 | 29 | 0.219 | ND^3^ | ND | ND |
| **6** |  | INNLNEKLEETNKEYTNLQNNYTNE | 22 | 16 | 0.201 | 50 | 12 | 0.107 | ND | ND | ND |
| **7** |  | IDKLNNEKGTLNSKISELNVQIMDL | 4 | 3 | 0.141 | 24 | 7 | 0.120 | ND | ND | ND |
| **8** |  | IKTMNTQISTLKNDVHLLNEQIDKLNNEKGTLNSKISELNVQIMDL | 70 | 43 | 0.294 | 36 | 26 | 0.274 | 15 | 5 | 0.101 |
| **9** |  | LLSKDKEIEEKNKKIKELNNDIKKL | 32 | 8 | 0.194 | 56 | 40 | 0.258 | 26 | 10 | 0.132 |
| **10** |  | LNLVDQGKKKLKKDVEKQKKEIEKL | 35 | 5 | 0.185 | 10 | 7 | 0.117 | ND | ND | ND |
| **11** |  | ICSLTTEVMELNNKKNELIEENNKLNLVDQGKKKLKKDVEKQKKEIEKL | 54 | 32 | 0.285 | 40 | 12 | 0.456 | 28 | 28 | 0.156 |
| **12** |  | VDKIEEHILDYDEEINKSRSNLFQLKNEICSLTTEVMELNNKKNELIEENNKLNLVDQGKKKLKKDVEKQKKEIEKL | 65 | 41 | 0.242 | 74 | 45 | 0.307 | 26 | 23 | 0.174 |
| **13** |  | LDENEDNIKKMKSKIDDMEKEIKYR | 27 | 27 | 0.214 | 38 | 24 | 0.149 | 64 | 26 | 0.170 |
| **14** | PFC0245c | GMNNMNGDINNIN(GDINNMN)4 | 41 | 30 | 0.266 | 40 | 21 | 0.185 | 44 | 31 | 0.215 |
| **15** | PFD0110w | LIKYMNERYQNMQQGYNNLTNYINQYE | 27 | 11 | 0.146 | 50 | 10 | 0.167 | ND | ND | ND |
| **16** |  | DINSTNNNLDNMLSEINSIQNNIHTYI | 35 | 3 | 0.121 | 31 | 17 | 0.163 | ND | ND | ND |
| **17** |  | EKKLDILKVNISNINNSLDKLK | 16 | 0 | 0.123 | 33 | 17 | 0.142 | ND | ND | ND |
| **18** |  | DVHNIKEDYNLLQQYLNYMKNEMEQLK | 57 | 32 | 0.210 | 38 | 7 | 0.100 | ND | ND | ND |
| **19** |  | DEKINDYLEEIKNEQNKIDKTIDDI | 27 | 5 | 0.136 | 38 | 10 | 0.148 | ND | ND | ND |
| **20** |  | MDVVINQLRDIDRQMLDLYKELDEK | 59 | 0 | 0.192 | 55 | 19 | 0.133 | ND | ND | ND |
| **21** |  | ISNIFKDIQNIKKQSQDIITNMNDM | 43 | 3 | 0.179 | 26 | 10 | 0.098 | ND | ND | ND |
| **22** |  | LEEIIKNLDILDEQIMTYHNSIDEL | 59 | 19 | 0.181 | 67 | 10 | 0.146 | ND | ND | ND |
| **23** |  | FQKVKEKAEIQKENIEKIKQEINTL | 24 | 19 | 0.203 | 7 | 7 | 0.134 | ND | ND | ND |
| **24** |  | EKKLDILKVNISNINNSLDKLKKYYEEALFQKVKEKAEIQKENIEKIKQEINTL | 32 | 30 | 0.267 | 14 | 12 | 0.121 | ND | ND | ND |
| **25** | PFD1115c | D(VTHLTND)4VTHLT | 0 | 0 | 0.153 | 98 | 12 | 0.134 | ND | ND | ND |
| **26** | PFE0570w | NLNKVKININDLNNNIVDVNNSIHNIE | 0 | 0 | 0.125 | 43 | 12 | 0.148 | 31 | 15 | 0.113 |
| **27** | MAL6P1.37 | KKRNVEEELHSLRKNYNIINEEIEEIT | 54 | 30 | 0.265 | 69 | 33 | 0.237 | 18 | 8 | 0.124 |
| **28** |  | YIDIKKKISELQKDNESLKIQVDRL | 22 | 3 | 0.102 | 69 | 10 | 0.113 | ND | ND | ND |
| **29** | MAL7P1.162 | QMEGFQKQLDRLSDSLSKIQKALGEYL | 3 | 0 | 0.122 | 24 | 10 | 0.129 | ND | ND | ND |
| **30** | PF11_0213 | LKLNEGLENIKQELHIIDRELKNIL | 16 | 5 | 0.126 | 45 | 7 | 0.128 | 23 | 3 | 0.138 |
| **31** | PFL1135c | NINSVNNNINSVDNNINNVDNNINSVN | 0 | 0 | 0.136 | 33 | 17 | 0.193 | 36 | 18 | 0.169 |
| **32** | MAL13P1.147 | NIIQIKNDIEQCQKSIKKIEDNLNTYE | 0 | 0 | 0.123 | 19 | 14 | 0.189 | ND | ND | ND |
| **33** | PF13_0277 | YIDDVDRDVENYDKGIANVDHHLNDVH | 19 | 8 | 0.125 | 10 | 7 | 0.121 | ND | ND | ND |
| **34** | MAL13P1.336 | NMNNMNNNMNNMNNNMNNNMNNMNNMN | 11 | 8 | 0.207 | 14 | 12 | 0.144 | ND | ND | ND |
| **35** | PF14_0045 | ARDDIQKDINKMESELINVSNEINRLD | 43 | 24 | 0.258 | 50 | 21 | 0.180 | ND | ND | ND |
| **36** | PF14_0093 | SSNNLSDQINILNNNIQHINSTFNNLR | 0 | 0 | 0.138 | 52 | 10 | 0.133 | ND | ND | ND |
| **37** | PF14_0175 | NNNVNNINMNNINSNVNNINNSMNNIN | 22 | 16 | 0.358 | 21 | 17 | 0.129 | ND | ND | ND |
| **38** | PF14_0089 | NITNINKNIENIKNDMSNLNNMNDSNQ | 41 | 14 | 0.163 | 17 | 12 | 0.107 | 28 | 8 | 0.135 |
| **39** | PF11_0210 | IEINMLTNNLLREMMKIKNKLQKLSNLLNALRSNIEKILKN | 59 | 11 | 0.118 | 29 | 0 | 0.093 | ND | ND | ND |
| **40** | PFC0235w | NEIKELNNTLNKYKEEMNNYKEEIIVINEKYKLLEIELCK | 59 | 24 | 0.161 | 48 | 17 | 0.149 | ND | ND | ND |
| **41** | PF11_0455 | RLINNIEEIYNSNCEQIQNVRDEFAELKNDLNKIMNLINI | 65 | 35 | 0.188 | 40 | 0 | 0.113 | ND | ND | ND |
| **42** | PF07_0014 | NSLDYYKKVIIKLKNNINNMEEYTNNITNDINVLKAHID | 54 | 11 | 0.155 | 48 | 10 | 0.150 | ND | ND | ND |
| **43** | PFD0685c | STDINSLNDEVKKLKEELNKIRNEYDDFKNKLELLYQK | 16 | 5 | 0.096 | 63 | 35 | 0.224 | 8 | 3 | 0.096 |
| **44** | MAL6P1.61 | IPLNQKVLEISKKLNNMNNNINEYKNYLSNFIHMLKE | 19 | 11 | 0.144 | 40 | 10 | 0.191 | ND | ND | ND |
| **45** | PF11_0207 | EEIKEEIKEVKEEIKEVKEEIKEVKEEIKEVKEEIKE | 70 | 57 | 0.554 | 62 | 48 | 0.263 | 59 | 36 | 0.295 |
| **46** | MAL13P1.176 | TIVQNSYNSFSDINKNINDIDKEMKTLIPMLDELLNE | 14 | 3 | 0.097 | 20 | 13 | 0.290 | 3 | 3 | 0.098 |
| **47** | PFA0635c | NHDTRINDYNKRLTEYNKRLTEYNKRLTEYTKRLNE | 3 | 0 | 0.093 | 28 | 8 | 0.199 | 21 | 3 | 0.110 |
| **48** | PF11_0240 | KGLEEANEKLQIVREKVQSLKAKLSELISQYDHAIY | 5 | 3 | 0.086 | 28 | 5 | 0.124 | ND | ND | ND |
| **49** | MAL6P1.80 | NNINNINNNINNINNNINNINNNINNINNNVNNYY | 22 | 0 | 0.108 | 15 | 0 | 0.118 | ND | ND | ND |
| **50** | PFL1605w | KNDINVQLDDINVQLDDINVQLDDINIQLDEINLN | 43 | 32 | 0.352 | 75 | 43 | 0.220 | 21 | 8 | 0.111 |
| **51** | PFD0985w | DNNVNNMDNNVNNVDNNVNNVDNNLNNVDNNVNN | 41 | 14 | 0.127 | 55 | 35 | 0.221 | 49 | 8 | 0.144 |
| **52** | PFL0770w | KIQIEEIKKETNQINKDIDHIEMNIINLKKKIEF | 51 | 24 | 0.180 | 36 | 24 | 0.187 | 26 | 13 | 0.108 |
| **53** | PFE0595w | MSQEKISEIIKDISALKTSCEKLNSQLDELITQ | 22 | 3% | 0.100 | 24 | 0 | 0.088 | ND | ND | ND |
| **54** | MAL6P1.147 | DSMNNHKDDMNNYNDNINNYVESMNNYDDIMNK | 59 | 43% | 0.288 | 76 | 36 | 0.260 | 31 | 18 | 0.199 |
| **55** | PF13_0065 | TSFSKYVRQLEQYFDNFDQDFLSLRQKISDILQ | 11 | 3 | 0.119 | 35 | 5 | 0.125 | ND | ND | ND |
| **56** | PF14_0013 | PYLRRAKHNLNNLQGGINNLYSSVNVVYDNLFN | 35 | 3 | 0.145 | 24 | 12 | 0.165 | ND | ND | ND |
| **57** | PF13_0198 | MEIKTIVQNSYNSFSDINKNINDIDKEMKTLI | 27 | 11 | 0.146 | 12 | 10 | 0.161 | ND | ND | ND |
| **58** |  | TISELEQEFNNNNQKLDNILQDINAMNLNINILQT | 35 | 22 | 0.130 | 40 | 25 | 0.143 | ND | ND | ND |
| **59** | PF14_0397 | SLLDTLEKSVKGIDENIEKYNKELNVIKQKIE | 19 | 11 | 0.143 | 24 | 14 | 0.162 | ND | ND | ND |
| **60** | PFC0810c | KNVIELKEYLEDLKKRMFDMQKRLNDIIITK | 27 | 14 | 0.152 | 29 | 19 | 0.175 | ND | ND | ND |
| **61** | MAL7P1.13 | SNKTFEKLNEKLNDIRNDVTNYKNELEEFKN | 14 | 8 | 0.121 | 14 | 5 | 0.130 | ND | ND | ND |
| **62** | PFL0150w | SSISSSLTNISSSLTNISSSLTNISSSLSNS | 5 | 0 | 0.101 | 53 | 18 | 0.156 | ND | ND | ND |
| **63** | PF14_0444 | NNEMDETINKLKKDINKLNEKIEKYDNFMKM | 11 | 8 | 0.126 | 29 | 12 | 0.139 | ND | ND | ND |
| **64** | MAL6P1.131 | NNFVNNKMNNMNNMKNNMNNMNNIMNNIMN | 22 | 14 | 0.141 | 24 | 14 | 0.178 | ND | ND | ND |
| **65** | PFB0460c | NNVIRSKMYNIKKRISKINDELHELSNFFL | 5 | 0 | 0.123 | 21 | 0 | 0.132 | ND | ND | ND |
| **66** | PFL0250w | MCELNVMENNMNNIHSNNNNISTHMDDVIE | 51 | 32 | 0.280 | 74 | 31 | 0.281 | 56 | 28 | 0.164 |
| **67** | PFL1235c | TYTLSKLNNQINELTKKINILRGNLDKARK | 19 | 5 | 0.144 | 12 | 5 | 0.122 | ND | ND | ND |
| **68** | PFL2310w | NFFLEQMENDMSSTYDKMNRINMDLSKLKR | 27 | 5 | 0.172 | 17 | 10 | 0.217 | ND | ND | ND |
| **69** | PF13_0088 | EKLVKHLDVIDKLIENIYDNINNLNEYINK | 22 | 14 | 0.167 | 21 | 5 | 0.122 | ND | ND | ND |
| **70** | PFB0765w | EKLNDMQKKLNDVNEKYKNIVECLNNYKT | 27 | 8 | 0.161 | 17 | 7 | 0.143 | ND | ND | ND |
| **71** | PFB0765w | KLEEMKQKNKELINNLNDISDELKNCIEQVNSVSRNMANVEK | 57 | 32 | 0.144 | 62 | 27 | 0.154 | ND | ND | ND |
| **72** | PFC0760c | EEIYKLNNDIDMLSNNCKKLKESIMMMEK | 24 | 14 | 0.188 | 21 | 5 | 0.147 | ND | ND | ND |
| **73** |  | KEIQMLKNQILSLEESIKSLNEFINNLKN | 30 | 11 | 0.127 | 67 | 45 | 0.201 | 26 | 3 | 0.103 |
| **74** | PFE0100w | TLIDSFNLNLSYLRESINNKKKHINKIND | 24 | 14 | 0.220 | 33 | 2 | 0.177 | ND | ND | ND |
| **75** | PFL2520w | EKLYILEKSINKLKKLLNDINNKYQTIKK | 8 | 3 | 0.140 | 14 | 0 | 0.146 | ND | ND | ND |
| **76** | MAL13P1.304 | GGLKNSNHNLNNIEMKYNTLNNNMNSINK | 57 | 27 | 0.353 | 50 | 36 | 0.259 | 51 | 10 | 0.136 |
| **77** | PF08_0048 | EKLKKYNNEISSLKKELDILNEKMGKCT | 54 | 43 | 0.309 | 79 | 4 | 0.359 | 51 | 36 | 0.192 |
| **78** | PF07_0021 | KNMDTVYKNIINMSNNMTQMYNSMNNMSHNIINASHDMMDASGNINSH | 41 | 14 | 0.159 | 53 | 20 | 0.143 | ND | ND | ND |
| **79** | PFB0315w | EKMNMKMEQMDMKMEKIDVNMDQMDVKMEQMDVKMEQMDVKMKRMNK | 76 | 51 | 0.176 | 93 | 65 | 0.406 | 54 | 13 | 0.109 |
| **80** | MAL8P1.12 | KNKLNKKWEQINDHINNLETNINDYNKKIKEGDSQLNNIQLQCENIEQKINKIKE | 89 | 57 | 0.297 | 80 | 48 | 0.246 | 33 | 5 | 0.101 |
| **81** | PF07_0086 | NEMNKEVNKMNEEVNKMNEEVNKMNEEVNKMNKEVNKMDEEVNKMNKEVNKMNK | 89 | 51 | 0.352 | 68 | 55 | 0.378 | 15 | 21 | 0.100 |
| **82** | MAL13P1.96 | EIINEIEKKIEDIEKNINITKENLKELENKITELQSSFSSYENEMKHVVKKIEDLEK | 27 | 14 | 0.144 | 70 | 48 | 0.218 | ND | ND | ND |
| **83** | PFC0345w | QNKMENDMNIIKNDMNIMENDMNIMENDMNIIKNDMNIMEKDMNIIKNDMNIIKNNMNIIKNEMNIIKNV | 51 | 46 | 0.302 | 75 | 73 | 0.505 | 15 | 5 | 0.107 |
| **84** | PFL0115w | DFLDVIYYKLNIKEINKSLTEVKNELTELQKNQEEAKNILAFK | 51 | 11 | 0.107 | 38 | 8 | 0.097 | ND | ND | ND |
| **85** | PFL0350c | ASIDNINKNINCINNDVDNINSNINNINDNIHKINSNVYGN | 32 | 24 | 0.240 | 75 | 43 | 0.163 | ND | ND | ND |
| **86** | PFL1930w | NFIKELELQIKNLNNEINTLNDMLKDSEEEIRMLNHTLEEK | 3 | 5 | 0.129 | 8 | 3 | 0.097 | ND | ND | ND |
| **87** |  | KYKIEINVLNDEITKLKNEINTYKNDLKNINATLDFYKST | 43 | 11 | 0.107 | 28 | 3 | 0.105 | ND | ND | ND |
| **88** | PF13_0120 | NVLEYAELIIDRQRDKINELEKKLEELRSSSEELQKNVIK | 30 | 8 | 0.099 | 35 | 8 | 0.114 | ND | ND | ND |
| **89** | PF13_0239 | GIFIYNMNLLREILKLMTDNIDTLKDKINEIKCSYAFLK | 38 | 5 | 0.100 | 20 | 3 | 0.089 | ND | ND | ND |
| **90** | PFD0520c | TKKLNKELSEGNKELEKLEKNIKELEETNNTLENDIKV | 59 | 41 | 0.401 | 88 | 53 | 0.406 | 28 | 10 | 0.128 |
| **91** | PF07_0111 | NFVNNYINENILNLKSVDNYLEKINNKIKDLDDNINDR | 19 | 3 | 0.129 | 20 | 0 | 0.130 | ND | ND | ND |
| **92** | MAL6P1.254 | PDFDAYNEKLGSISQSIDEIKKKIDNLQKEIKVANK | 41 | 5 | 0.127 | 30 | 19 | 0.198 | ND | ND | ND |
| **93** | PF11_0424 | QLEEKTKQYNDLQNNMKTIKEQNEHLKNKFQSMGK | 38 | 8 | 0.131 | 50 | 30 | 0.203 | ND | ND | ND |
| **94** | PFD0970c | ENINNMDEKINNVDEQNNNMDEKINNVDEKK | 43 | 43 | 0.200 | 78 | 50 | 0.319 | 10 | 3 | 0.107 |
| **95** | PF14_0574 | EKGLKSLNEKIKNYDSIIEEQKNQLENLKM | 35 | 22 | 0.216 | 70 | 38 | 0.188 | ND | ND | ND |

^1^ % of positive responses evaluated as OD values higher than the mean negative control + 3SD.

^2^ OD ratio higher than 2 between the mean duplicate experimental and mean negative control OD.

^3^ Not Done

^4^ A, alanine; C, cysteine; D, aspartic acid; E, glutamic acid; F, phenylalanine; G, glycine; H, histidine; I, isoleucine; K, lysine; L, leucine; M, methionine; N, asparagine; P, proline; Q, glutamine; R, arginine; S, serine; T, threonine; V, valine; W, tryptophan; Y, tyrosine
