# Supplementary material for: Rapid Identification of Malaria Vaccine Candidates Based on α-Helical Coiled Coil Protein Motif
Source: PLoS One. 2007 Jul 25;2(7):e645. doi: 10.1371/journal.pone.0000645 (PMC1920550; doi:10.1371/journal.pone.0000645)
Supplement: Table S3 — Structural feature and cellular location prediction of the proteins containing the peptides whose specific antibodies were tested in ADCI (Table 1). (0.05 MB DOC) [file pone.0000645.s005.doc]

| **8, 9, 11, 12, 13** ^a^ | PFB0145c ^b^ | TM (1) ^c^ | Cytoplasm ^d^ | TM (1) | 5 (446, 1082, 1403, 1540, 1833) ^e^ |
| --- | --- | --- | --- | --- | --- |
| **14** | PFC0245c | TM (9) | Peroxysomes | TM (13) | 6 (752, 1447, 1502, 1957, 2933, 3239) |
| **27** | MAL6P1.37 | No | Cytoplasm | No | 1 (98) |
| **45** | PF11_0207 | TM (1) | Cytoplasm | TM (1) | 0 |
| **50** | PFL1605w | No | Cytoplasm | No | 3 (170, 252, 1064) |
| **52** | PFL0770w | TM (2) | Mitochondria | SP, TM (2) | 4 (131, 151, 355, 406) |
| **54** | MAL6P1.147 | TM (20) | Cytoplasm | TM (20) | 25 ^f^ (935, 10226) |
| **66** | PFL0250w | TM (2) | Cytoplasm | TM (2) | 1 (243) |
| **72** | PFC0760c | TM (1) | Cytoplasm | TM (1) | 11 ^f^ (293, 2418) |
| **76** | MAL13P1.304 | No | Nucleus | No | 4 (827, 1108, 1363, 1583) |
| **77** | PF08_0048 | TM (2) | Cytoplasm | TM (2) | 3 (1285, 1871, 1998) |
| **79** | PFB0315w | TM (1) | Cytoplasm | TM (1) | 5 (185, 1227, 1380, 1399, 1404) |
| **80** | MAL8P1.12 | No | Cytoplasm | No | 5 (278, 334, 433, 658, 743) |
| **81** | PF07_0086 | TM (7) | Cytoplasm | TM (7) | 4 (979, 1211, 3296, 3319) |
| **83** | PFC0345w | TM (2) | Cytoplasm | TM (1) | 2 (8, 221) |
| **90** | PFD0520c | No | Cytoplasm | No | 0 |
| **94** | PFD0970c | No | Cytoplasm | TM (1) | 4 (1386, 2255, 2614, 2940) |

^a^ Peptide number

^b^ Analyzed sequences were taken from PlasmoDB5.2

^c^ TM, transmembrane regions, number of predicted TMs is in brackets, prediction was made by using TMpred program (K. Hofmann & W. Stoffel (1993) Biol. Chem. Hoppe-Seyler 374,166.

^d^ Prediction is done by pTARGET (Guda C, Subramaniam S. Bioinformatics. 2005 21,3963) with confidence ranging from 80 to 100%.

^e^ Number of predicted PEXEL motifs. The amino acid positions are in brackets. Prediction was done using the following pattern [KR][GAVLIMFWPSTCYNQ][LIA][GAVLIMFWPSTCYNQ] [DEQ] [21, 22].

^f^ For these proteins only the amino acid positions of the first and the last PEXEL motifs are given.
