# Supplementary material for: Rapid Identification of Malaria Vaccine Candidates Based on α-Helical Coiled Coil Protein Motif
Source: PLoS One. 2007 Jul 25;2(7):e645. doi: 10.1371/journal.pone.0000645 (PMC1920550; doi:10.1371/journal.pone.0000645)
Supplement: Supplementary file 4 [file pone.0000645.s004.doc]

Peptide	Human purified Abs																		α-27 mouse sera
	α-8	α-9	α-12	α-13	α-14	α-27	α-45	α-52	α-54	α-66	α-76	α-77	α-79	α-80	α-81	α-83	α-90	α-94	
8	3.4	5.3	1.0	0.8	0.8	1.0	1.0	0.9	1.0	0.8	1.0	1.0	0.8	0.6	0.7	1.1	0.7	0.8	
9	0.8	6.6	1.0	1.2	0.9	0.8	0.8	0.7	0.7	0.9	0.9	1.0	0.6	0.6	0.6	0.6	0.6	1.0	0.9
12	1.0	1.4	13.2	0.8	1.1	2.8	3.1	1.1	1.0	1.1	1.6	3.6	1.1	3.3	1.9	0.9	0.8	0.7	0.9
13	2.1	0.6	1.0	11.4	0.7	0.8	0.8	0.9	1.0	0.7	1.1	0.8	0.7	0.6	0.6	0.7	0.6	0.6	
14	0.9	0.8	1.1	0.9	4.2	0.9	0.9	1.1	3.7	1.2	8.2	1.1	0.8	2.6	1.3	0.8	0.6	1.2	
27	0.9	0.9	0.9	0.8	0.8	10.9	4.8	2.7	1.0	0.7	1.0	0.7	0.6	0.6	0.7	0.6	0.5	0.6	7.9
45	0.9	1.3	3.0	1.1	0.7	12.2	18.2	3.9	1.0	0.8	1.2	1.1	0.7	0.6	0.6	0.7	0.6	0.6	1.0
52	1.0	0.8	0.8	0.7	1.5	1.4	1.2	8.5	0.8	1.2	1.3	1.0	0.7	0.6	0.6	0.7	0.6	0.7	
54	1.0	1.9	1.0	0.9	2.0	0.9	0.8	2.0	18.6	0.9	1.8	0.8	0.9	0.9	0.7	0.8	0.8	1.5	
66	1.3	0.9	0.8	0.8	1.7	1.3	1.0	0.8	1.3	3.3	1.8	1.0	0.7	0.6	0.6	0.7	0.6	0.7	
76	0.9	0.9	0.8	0.8	1.3	1.1	0.8	0.9	2.7	1.1	9.6	1.1	0.8	0.7	0.6	0.9	0.6	0.8	
77	1.2	1.1	0.8	0.8	1.1	1.3	1.1	2.6	0.8	1.6	1.4	8.9	0.8	0.7	0.7	0.8	0.6	0.7	
79	1.0	0.9	1.2	1.5	1.1	0.8	0.7	0.7	0.7	0.8	1.1	0.9	3.2	0.7	1.1	0.9	0.7	0.9	
80	0.7	0.8	0.7	0.6	0.7	1.5	0.6	0.8	1.2	0.7	1.0	0.6	0.7	8.1	2.7	0.7	0.6	1.0	
81	0.7	1.2	0.7	0.8	0.9	0.7	0.6	1.0	0.7	0.8	2.7	0.8	0.6	1.0	11.6	0.6	0.9	0.7	
83	0.7	1.1	0.7	0.8	0.9	0.7	0.6	2.5	2.1	0.7	1.2	0.8	0.6	0.8	1.4	6.0	0.6	0.6	
90	0.7	1.4	1.1	1.1	0.9	1.0	0.8	0.7	0.7	0.9	1.1	1.0	1.2	0.7	1.3	1.1	4.5	1.0	
94	0.9	0.8	0.9	1.1	0.8	1.0	0.8	0.9	1.3	0.9	1.3	1.0	1.0	0.8	1.0	1.1	0.7	6.0	
